# How are Immigrant Children in Sweden Faring? Mean Income, Affluence and Poverty Since the 1980s

**DOI:** 10.1007/s12187-016-9416-9

**Published:** 2016-09-08

**Authors:** Björn Gustafsson, Torun Österberg

**Affiliations:** 1 Department of Social Work, University of Gothenburg, P.O. Box 720, SE 405 30 Gothenburg, Sweden; 2 Institute for the Study of Labor (IZA), Bonn, Germany

**Keywords:** Sweden, Immigrants, Poverty, Affluence, Income

## Abstract

This article presents new research on income-based child indicators for immigrant children from 17 different national backgrounds and children of parents born in Sweden observed during the 3-year periods 1983–85, 1995–97 and 2008–10. This research examines mean household income, representation at the top of the income distribution and relative poverty differ for immigrant children from the corresponding levels among children with native born parents. Most of the analysis is concentrated on the second generation of immigrant children. It is shown that the relative position of immigrant children deteriorated between 1983–85 and 1995–97 when the labour market situation of immigrant parents weakened more than among native born parents. Changes thereafter were more complex. Children born in Sweden to parents from Denmark, Norway or Germany were as likely as children of native born parents to be observed at the top of the income distribution in contrast to children of parents from countries with middle or low human development. Poverty rates among immigrant children were higher among all categories of immigrant children in 2008–10 than among children of native born parents. These cross origin differences in income-based child indicators can be attributed to the reasons and qualifications parents had when they entered Sweden and the number of years since their immigration. A majority of children living in Sweden that are classified as poor in 2008–10 were immigrant children of various categories.

## Introduction

Many countries with high Human Development have received waves of immigration originating from countries with lower Human Development. Such immigrants and their children often differ from the non-immigrant population in appearance, name, religion and language, thus their integration into the new country has attracted much policy concern. From this follows an interest in developing and measuring child indicators that make it possible to compare immigrant and non-immigrant children along several dimensions.^1^ This article aims to contribute to the literature on child indicators by reporting new research on the Swedish case focusing on income-based child indicators. This research has a time dimension as we study the 3-year periods 1983–85, 1995–97 and 2008–10.^2^

The Swedish case is interesting as the country has long been well known for its ambitious welfare programmes, comparably equal distribution of income and low relative poverty rates. In European comparisons of well-being for the average child, Sweden ranks higher than a majority of the European states (Bradshaw and Richardson 2009). However, since the beginning of the 1990s there have been substantial changes in Swedish society affecting the income generating process. Rates of unemployment are no longer uniquely low, having since the 1990s been similar to those observed in many other Northern European countries. From the end of the 1990s to 2007 workers’ real earnings increased rapidly while the real value of many transfers received by persons of active work age changed little. Furthermore, changes in the tax code made taxes less progressive and lowered the tax burden for wage earners but not for households living on transfers. All those changes contributed to widen the income gap between full-time workers and others of active work age, making the distribution of household income less equal. As shown in Table 1 did the Gini coefficient for disposable income increase from 0.21 for the 3 year accounting period 1983–85 to 0.29 for the accounting period 2008–10.

**Table 1:** Unemployment rate, median income and Gini coefficients for the three periods studied

Years / Variable	Unemployment rate (%)	Median 3-year income prices of 2011	Gini coefficient for household 3-year disposable income	Gini coefficient for household 3-year factor income
		’000s of SEK		
1983–85	3.3	117.3	0.21	0.42
1995–97	9.4	143.4	0.23	0.46
2008–10	7.7	209.2	0.29	0.48

Sources: Unemployment rate from Eurostat, http://appsso.eurostat.ec.europa.eu/nui/show.do?dataset=une_rt_a&lang=en, and authors’ calculations based on data presented in the text. Prices of 2011

Sweden is also known during more recent years for having received a large inflow of migrants, particularly such coming for political reasons or relatives to such persons. As a consequence, not fewer than 462 000 persons aged under 18 had some kind of immigrant background, that is, had immigrated themselves or had at least one parent who had immigrated.^3^ Immigrant children in 2010 made up 28 % of all children living in Sweden. This percentage was higher than that in the Netherlands and France (both 17 %) as well as the United Kingdom (16 %), was similar to the level in Germany (26 %), but lower than that in Switzerland (39 %) and Australia (33 %) (Hernandez 2010). The single largest sub-category of immigrant children in Sweden is those who are born in Sweden to two foreign-born parents, the second generation. In 2010 they numbered 194 000, or 16 % of all children living in Sweden.^4^ It is child indicators based on the income situation in said households that are the focus of this article. Immigrant children living in Sweden have diverse country backgrounds and their parents entered Sweden for many different reasons. An increasingly large proportion of immigrant children in Sweden have a background in countries that rank low or have middle standing on the Human Development Index, typically located outside Europe. There are a number of reasons why immigrants have difficulties finding employment. The difficulty in finding employment means that the gap in employment rates between immigrants and non-immigrants in Sweden has become one of the largest among countries that rank high on the HDI (Dustman and Frattini 2011; de la Rica et al. 2015).

This article aims to answer the following research question: How and why have changes in the Swedish labour market and its welfare system together with the changed country composition of immigrant children led to changed immigrant native gaps in household income–based child indicators? We present new research for the second generation of immigrant children from 17 different national backgrounds and for non-immigrant children. We look at the mean child income, representation at the top of the income distribution as well as relative poverty. In many countries researchers typically have to base their studies of income among households with immigrant children on sample surveys including few or relatively few immigrant children and often there are problems with non-response in the surveys. However, as we have been able to work with income information from tax records and transfers received for all persons registered as living in Sweden we are in these respects in a better position. From our data we compute mean income, measures of representation at the top of the income distribution and relative poverty rates for each of the 17 categories of second generation immigrant children and the corresponding levels for children with native born parents.

Several authors have investigated how and why the labour market situation of immigrants to Sweden has deteriorated during recent years and others poverty among adult immigrants.^5^ However, those studies are silent on average income, affluence and poverty seen from the perspective of immigrant children and natives as well as on how they have changed. True, there are some previous studies on poverty among immigrant children in Sweden, but none exist on mean child income or on the representation at the top of the income distribution.

Certain previous studies of child poverty in Sweden do not focus on the immigrant aspect. One such example is Mood and Jonsson (2015) who investigated trends in child poverty and showed that the offspring of immigrants more often experience economic hardship than those of natives. Another example is Lindquist and Sjögren Lindquist (2012) who analysed dynamic aspects of child poverty using the LINDA panel (a register-based longitudinal data set) for the years 1991 to 2004. In a probit analysis on the risk of being permanently poor, the immigrant status of the parent was found to be positively related to poverty status. In addition they found that short education and the risk of being permanently poor were positively related. The work of Galloway et al. (2015), who for the period 1993 to 2001 compared the development and dynamic aspects of child poverty in Denmark, Norway and Sweden, is closer to this study. Those authors reported that immigrant children constituted an increasingly larger proportion of the poor children in all three Scandinavian countries. They also showed that child poverty rates are generally high on arrival in the new country and typically decline with the passing years since immigration. The present study covers a longer period, it deals with mean child income as well as representation at the top of the income distribution and it defines 17 countries of origin. Gustafsson and Österberg (2015) studied the same years as here using the same database but limited their focus to children with parents born in Turkey and surrounding countries.

The rest of the article is laid out as follows: The next section provides a brief description of how Swedish macroeconomics and the welfare state have changed since the beginning of the 1980s. Section 3 describes how immigration to Sweden has resulted in an increasingly larger and also changed country of origin population of immigrant children. The child indicators we use are presented in Section 4, which also describes the data for the study. Section 5 provides results on mean child income for the 17 country of origin categories and children with native born parents referring to the three periods. Sections 6 and 7 supplements the analysis by providing information, respectively, on rates of affluence and relative child poverty rates. Finally we summarise and discuss our findings in Section 8.

## The Changed Swedish Scene

This study covers a period of two and a half decades, or approximately one generation. Data availability was a motivation for the start period 1983 to 1985 and the end period 2008 to 2010. In order to understand when most of the possible changes had occurred we chose to include the period 1995 to 1997 in the study, too.

The years 1983 to 1985 represent the ‘golden days’ of the Swedish welfare state. Unemployment was at that time experienced by a much lower proportion of the workforce than in almost all comparable countries, and, as a consequence, it was relatively easy for recent immigrants to find a first job (see Table 1). Wage dispersions were small from an international perspective and the income tax system had a rather redistributive structure. For decades, welfare programmes had been on a trajectory of expansion in terms of both eligibility criteria and benefit levels. Inequality in the distribution of income at the household level had for decades been on a downward path and the Swedish distribution of income had become one of the most equal among high income countries (see, for example, Smeeding et al. 1990).

However, this description has become increasingly inaccurate.^6^ One of the first changes that occurred in the early 1980s was the centralised wage negotiations between trade unions and the employer’s confederation that had taken place for decades being abolished. Thereafter wage inequality started slowly to increase. The income tax system was reformed in 1990 and 1991 with the purpose of decreasing its distortive consequences. The reform broadened the tax base, reduced progressivity in tax scales and introduced a separate tax scale for capital income. As a side effect the tax system became less redistributive. When the world’s economic downturn at the beginning of the 1990s reached Sweden it had very large consequences. In November 1991 the Swedish Central Bank was forced to abandon the fixed exchange rate. Keeping inflation, not unemployment, low became the prime economic policy goal. Another change in the policy regime framework was that Sweden in 1991 applied to become, and in 1995 became, a member of the European Union. Sweden’s GDP decreased in each of the years 1991, 1992 and 1993. Many workers left the labour force and unemployment increased to levels not previously experienced by those in the active generations. Initially, increased payments of unemployment compensation and reduced income taxes dampened the consequences of the earnings losses. However, those mechanisms also contributed to very rapidly increasing public sector deficits that soon had to be counteracted by tax increases as well as by cuts in public expenditure: The number of government employees reduced and many benefits became less generous.

Some of the consequences of these huge changes between 1983–85 and 1995–97 are shown in Table 1: The unemployment rate almost tripled, particularly affecting foreign-born workers, along with school-leavers.^7^ While it is true that median household income was 15 % higher in 1995–97 than in 1983–85, the increase was unequally shared, as indicated by the increase in the Gini coefficient for household disposable income, which moved from 0.21 to 0.23, or by 0.02 units. Still, this increase is somewhat smaller than when we compare Ginis computed for household factor income, which went up from 0.42 to 0.46, or by 0.04 units.^8^ The somewhat different changes in the two Ginis indicate that transfers and taxes to some extent dampened inequality increasing impulses coming from the markets.

From 1994 until 2007 Sweden experienced increased GDP in all years. It was when the great recession in export-countries hit Sweden in the autumn of 2008 that GDP fell. The downturn in 2009 was deep, but was quickly followed by a roughly equally large increase, meaning that the GDP averaged over 2008–10 was similar to the level in 2007. Unlike during the 1980s, real wages grew rapidly during most of the 2000s, benefiting households with working members. Median household income increased from 1995–97 to 2008–10 by as much as 46 %, or approximately two times as much as between 1983–85 and 1995–97 (see Table 1). However, it was not until the end of the 1990s that the employment situation improved, and then it rose only to levels substantially lower than before the rapid increase at the beginning of the 1990s. Although the levels of many benefits slightly increased at the end of the 1990s, further increases did not follow during the decade of rising earnings.

One policy goal of the conservative-liberal government that was in office from 2006 to 2014 was to increase the incentives for market work. One measure was to introduce, in a stepwise manner, earned income tax credits for wage earners while transfer receivers (including pensioners) did not benefit from those changes. A consequence of this is that, in some years, wage earners in Sweden pay less income tax than persons receiving equally large transfer payments. Other policy changes meant tightening eligibility criteria for various benefits (sickness benefits, unemployment benefits, disability pension, etc.). The changes in the transfer systems and income tax system contributed to making the distribution of income in 2008–10 more unequal than in 1995–97.^9^ Table 1 shows that while the Gini coefficient for factor income increased by 0.02 units from 1995–97 to 2008–10, the Gini coefficient for disposable income increased from 0.23 to 0.39, that is, by 0.06 units. This means that the redistributive property of transfers and income taxes had become smaller over time.

The development of household income during the first years of the new century meant that real income at the lower part of the income distribution grew only slowly while increases were large in the middle and at the top. Following from this is the fact that an increasingly larger proportion of the population live in households with income lower than 60 % of the contemporary median income (a commonly used criterion for the risk of being poor in EU countries). According to Statistics Sweden, such often used relative poverty rates stood at 9.9 % in 1995 but had climbed to as high as 17.3 % in 2010. Such a development means that, in international comparisons, Sweden in 2012 no longer stands out as having uniquely low relative poverty rates, see OECD (2015).

## Immigration to Sweden and the Number of Immigrant Children

During the first decades of the 1900s Sweden was a country with very few foreign-born inhabitants. However, as a consequence of a large influx of immigrants, the proportion of foreign-born persons steadily increased from 4.0 % in 1960 to 7.5 % in 1980, 11.3 % in 2000 and 14.7 % in 2010. Most foreign-born inhabitants arrive as young adults, many have a foreign-born partner and their children, born in Sweden, make up the second generation of immigrant children.

**Fig. 1:**
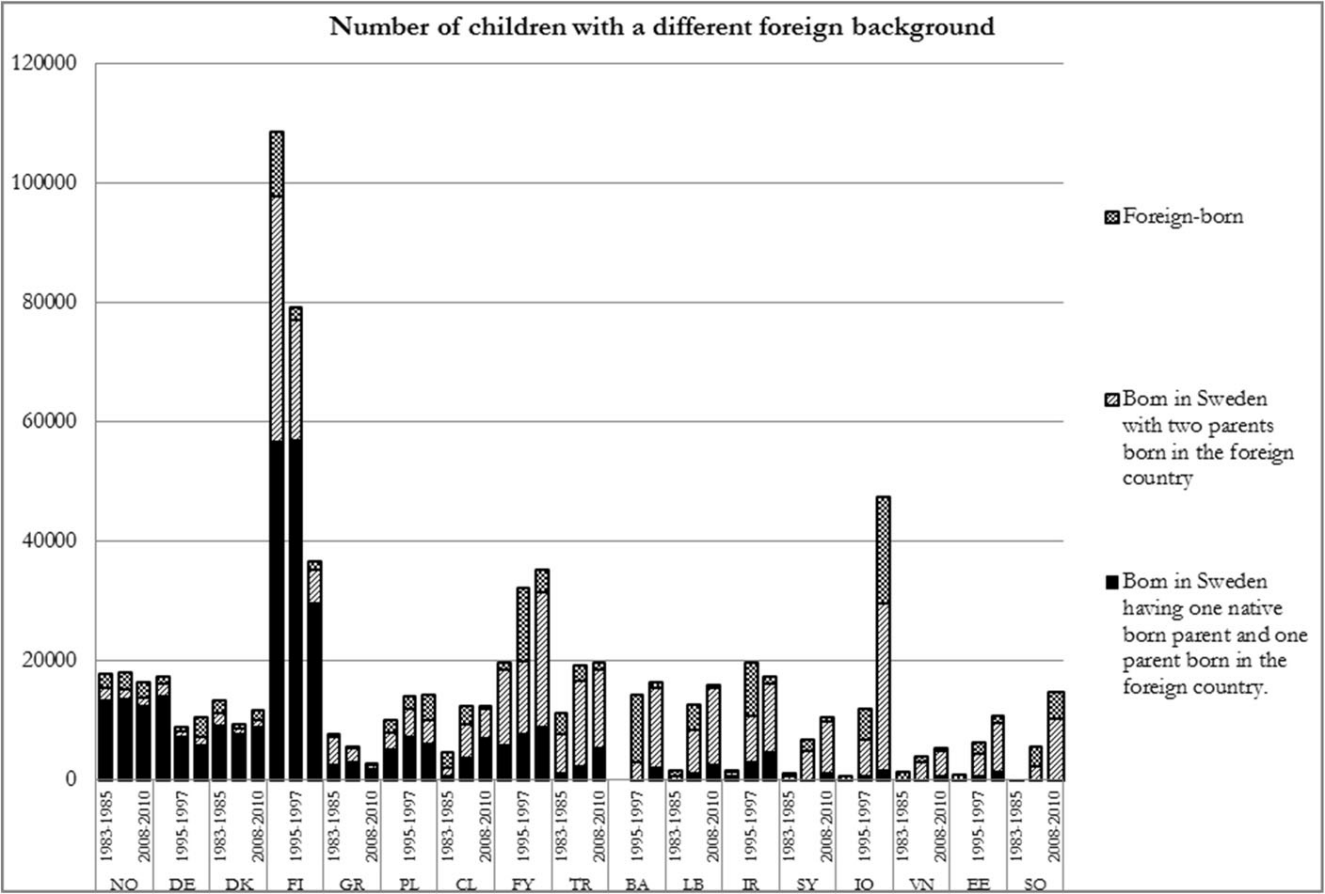
Number of children with a different foreign background. Source: Authors’ estimates based on data presented in the text. Countries are ranked after the Human development Index. Abbreviations: *NO* Norway, *DE* Germany, *DK* Denmark, *FI* Finland, *GR* Greece, *PL* Poland, *CL* Chile, *FY* Former Yugoslavia, *TR* Turkey, *BA* Bosnia and Herzegovina, *LB* Lebanon, *IR* Iran, *SY* Syria, *IQ* Iraq, *VN* Vietnam, *EE* Ethiopia and Eritrea, *SO* Somalia

Migrants have gone to Sweden for a number of different reasons, including to work, as refugees and for family reasons. The country of origin has changed considerably. In broad terms the immigrant waves have shifted from work migrants originating from countries like Finland and Yugoslavia arriving mainly during the 1960s and 1970s towards refugees and their relatives originating from a spectrum of countries: Chile (during the 1970s and the 80s), countries in the Middle East (from the 1980s onwards), Bosnia (during the 1990s) and Somalia (since the 1990s). For many years there have also been substantial migration flows for work reasons or for family reasons from countries with high HDI like Denmark, Germany and Norway. A particular characteristic of foreign-born persons from countries with high human development living in Sweden is that many have a native-born partner, and their children born in Sweden thus have a mixed background, as can be seen in Fig. 1.

How the migration flows together with how partnership is formed has resulted in an immigrant child population in Sweden, as is shown in Fig. 1 for 17 countries of background for the three periods studied.^10^ The figure is produced based on the data presented in the next section. The analysis in the following sections of the article concentrates on the second generation immigrant children with a background in those 17 largest countries of origin. Figure 1 also puts their number into the perspective of other categories of immigrant children: those who are themselves foreign born (in most cases a relatively small category) and children born in Sweden having one foreign-born parent and the other born in Sweden (a mixed background).

Several observations can be made from Fig. 1. First we can note a substantial change in country of origin across the years consistent with the description above of the different migration streams. In 1983–85 Finland was the dominating country of origin, as more than 100 000 children, approximately the size of one birth cohort of children living in Sweden, had such a background. Of those, approximately 50 000 were born in Sweden with both parents having been born in Finland and approximately the same number were of mixed background. As the children aged, the stream of migration from Finland dried up and some migrants and their families returned to Finland the number of immigrant children with a Finnish background fell to fewer than 40 000 in 2008–10.

In 2008–10 the largest country of background among immigrant children in Sweden was Iraq, with 48 000, of whom 28 000 were born in Sweden to two foreign-born parents, 18 000 were themselves born in Iraq and not more than 2 000 were of mixed background. The development towards a more diversified country of background composition among second generation immigrant children living in Sweden can be expressed by looking into the number of countries with at least 10 000 children belonging to the second generation (see Fig. 1). In 1983–85, as in 1995–97, there were three such countries of background: Finland, Yugoslavia and Turkey. In 2008–10 there were six: Iraq, Yugoslavia, Iran, Bosnia, Turkey and Somalia, but not Finland.

In some of the later sections of the article we will make comparisons between children born in Sweden to parents born in Bosnia, Somalia and Turkey. As we will see, children belonging to those categories were similar in 1995–97 according to mean average household income. However, thereafter the development was rather different. A large majority of parents born in Bosnia, most of whom arrived during the first part of the 1990s, gained a foothold in the Swedish labour market, and the income of the category improved remarkably rapidly. The corresponding development among the Somali and Turkish groups was much slower.^11^

## Concepts and Their Measurements

Child indicators can cover many aspects of life and be useful for policy-making in many spheres, and the information can be collected in different ways.^12^ This article focuses on household income-based child indicators that are central for making policies on taxes and transfers. The indicators used here take the disposable income in the household in which the child lives as the point of departure. To consider the composition of the household we adjust it by applying an equivalent scale.^13^ We then assign this adjusted household income to each person within the household; child as well as adult. Such an analytic choice is now standard in studies of the distribution of household income. In order to even out short-term fluctuations in income at the household level we average household income over a 3-year period at the household level and carry out the analysis for each of the three periods 1983–85, 1995–97 and 2008–10.^14^

We use data from Statistics Sweden originating from different registers. It refers to *all* persons who are registered as residing in Sweden and thus does not include asylum seekers as long as they have such status.^15^ It ought to be noted, too, that our data is not a sample. For the children (defined as persons aged under 18) and their parents we obtained information on demographic variables like year of birth, country of birth, number of years since immigration and place of domicile in Sweden from the population register. Information on the level of education of the parents used in the multivariate analysis originates from the register of education, which includes detailed administrative records of education completed in Sweden and information on education received outside Sweden obtained from questionnaires or validated certificates. The information on disposable income is derived from the income and tax register, which in turn receives its information from the tax authority and various authorities paying transfers to the households. Disposable income includes earnings, capital income (for example, interests and dividends), realised capital gains from selling stocks and real estate, and public sector transfers (pensions, sickness and unemployment benefits, parental benefits, et cetera). Disposable income is measured net of income taxes.

## Mean Child Income Among Second Generation Immigrant Children from 17 Countries and Native Children

In this section we report on mean income among second generation immigrant children from 17 countries as well as children having native born parents for the three periods 1983–85, 1995–97 and 2008–10. We are comparing cross-sections, which mean that no single individual who was classified as a child in 1983–85 is classified as such in 2008–10. The information is shown in Table 2 where we express the means in constant prices and, for an immigrant category, as a fraction of the value for the majority. We also report percentage changes for each of the two sub-periods. We have arranged the foreign countries in the table after the level of HDI (human development index) as observed in 2010.^16^ Several observations can be made from Table 2. First, the gaps in average child income towards the majority were in 1983–85 rather small for children with a background in countries with high human development. In contrast, gaps ranging up to 25 % were observed for immigrant children from countries with low or middle human development.

**Table 2:** Average equivalent disposable income among native children and children from 17 different origins 1983–85, 1995–97 and 2008–10

Parental country of birth	Average income 1983–85 SEK	Average income 1995–97 SEK	Average income 2008–10 SEK	Average income 1983–85as a fraction of native income	Average income 1995–97 as a fraction of native income	Average income 2008–10 as a fraction of native income	Growth rate 1995–97 compared to 1983–85 (%)	Growth rate 2008–10 compared to 1995–97 (%)
Sweden	113589	148362	235733	100 %	100 %	100 %	31 %	59 %
Norway	115532	132790	216953	102 %	90 %	92 %	15 %	63 %
Germany	118035	135547	212084	104 %	91 %	90 %	15 %	56 %
Denmark	115025	143202	221260	101 %	97 %	94 %	24 %	55 %
Finland	110954	135187	208024	98 %	91 %	88 %	22 %	54 %
Greece	104682	112918	192869	92 %	76 %	82 %	8 %	71 %
Poland	112085	126710	168662	99 %	85 %	72 %	13 %	33 %
Chile	103077	115816	164829	91 %	78 %	70 %	12 %	42 %
Yugoslavia	111165	112081	158419	98 %	76 %	67 %	1 %	41 %
Turkey	94653	98615	146800	83 %	66 %	62 %	4 %	49 %
Bosnia		93211	180836	0 %	63 %	77 %		94 %
Lebanon	85565	94412	138134	75 %	64 %	59 %	10 %	46 %
Iran	91352	108134	173256	80 %	73 %	73 %	18 %	60 %
Syria	94131	93976	134078	83 %	63 %	57 %	0 %	43 %
Iraq	89354	99865	132401	79 %	67 %	56 %	12 %	33 %
Vietnam	93966	108685	149164	83 %	73 %	63 %	16 %	37 %
Etiopia, Eritrea	100414	106784	154571	88 %	72 %	66 %	6 %	45 %
Somalia		99537	123472		67 %	52 %		24 %

Source: Authors’ calculations based on data presented in the text. Prices of 2011

However, the development in average income from 1983–85 to 1995–97 differed for children with native born parents and immigrant children. Mean income among majority children increased from 114 000 SEK per person and year to 148 000 SEK in 1995–97, or by 30 % (which is somewhat higher than for the entire population of Sweden as reported in Table 1). Immigrant children with parents from countries with high human development experienced lower income growth than majority children, and as a consequence an income gap appeared. Also, income among immigrants from countries with low and middle human development grew less rapidly than among children with native born parents; as a consequence their income gap towards the majority widened to become more than 30 % among those from Turkey, Lebanon, Syria and Iraq as for the new categories of Bosnia and Somalia.

Mirroring the change in Sweden’s GDP, mean child income grew more rapidly during the second sub-period than during the first for all categories of children. For children with native born parents, the growth was 59 %, or about twice as large as during the first sub-period (and somewhat larger than for the entire population of Sweden, as shown in Table 1). The average income of children with both parents having been born in Finland increased by 54 %, for those with parents born in Turkey by 49 % and for those whose parents were born in Yugoslavia by 41 %. As a consequence, the income gap towards children with native born parents increased slightly for those categories as was also the case for ten other categories of immigrant children. The opposite development took place among not more than four other categories of immigrant children, most notably the income increase for children with a background in Bosnia was as high as 94 % and propelled by the fact that many parents born in Bosnia found a job. Pay also attention to that the immigration stream from Bosnia was to a large extent concentrated to a short period of time in the 1990s.^17^ As a consequence, the gap in mean income towards native children decreased from 37 to 23 %. However, such a development did not occur in the categories having parents born in Turkey or among those having parents born in Somalia.

An interesting issue is: In case we widen the view to also observe children born in Sweden with one foreign born parent the other parent native born, to what extent do we find a similar picture? Table A1 in the Appendix shows that much of the description of the situation among second generation children and it’s changes across time also applies to the category of children with mixed background. However, in many but not all cases is the gap in average child income somewhat smaller for children with one native born parent and one foreign born parent compared to children who have two parents from the same foreign country. Another difference is that for the development from 1995–97 to 2008–10 among the mixed categories was somewhat different as the examples of an income growth more rapid than among native born were as common as examples on the contrary.^18^

## Rates of Affluence

**Fig. 2:**
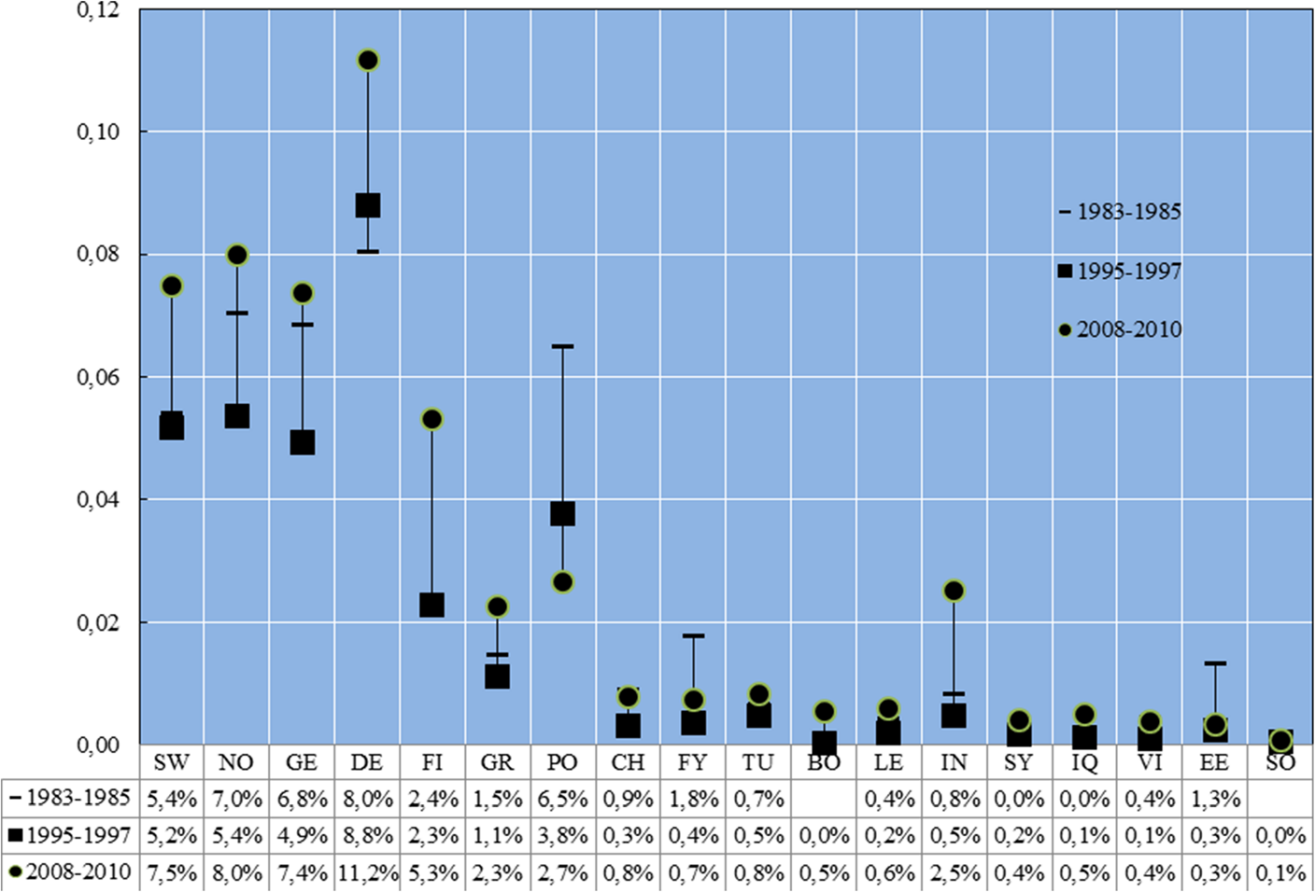
Representation at the top of the distribution of household equivalent disposable income among children from 17 different origins and native children 1983–85, 1995–97 and 2008–10 (percent). Source: Authors’ estimates based on data presented in the text. Countries are ranked after the Human development Index. Abbreviations: *SW* Sweden, *NO* Norway, *DE* Germany, *DK* Denmark, *FI* Finland, *GR* Greece, *PL* Poland, *CL* Chile, *FY* Former Yugoslavia, *TR* Turkey, *BA* Bosnia and Herzegovina, *LB* Lebanon, *IR* Iran, *SY* Syria, *IQ* Iraq, *VN* Vietnam, *EE* Ethiopia and Eritrea, *SO* Somalia

The rate of affluence is here defined as the proportion of the category that has a disposable equivalent income high enough to place the person in the top decile of the distribution of disposable income among all persons living in Sweden the same year. With this definition in mind, the proportion of children with native born parents in the top decile amounted to 5, 5 and 8 %, respectively, for the three periods studied. Figure 2 starts with rates of affluence for native children and thereafter orders countries according to HDI. There are no confidence intervals as the proportions are based on computations for all children living in Sweden, and are not estimates derived from a sample.

We see from Fig. 2 that children with parents born in countries with high HDI – Norway, Germany and Denmark – were all well represented at the top of the income distribution, as were the children of native born parents. This applied also during the first period under study to immigrant children from Poland.^19^ Another change across the years is the increased proportion of Finnish children found among the affluent. Figure 2 also shows that it is rather unusual for immigrant children with a background in a country with low or medium human development to be represented among the affluent in the population living in Sweden, and this is true for all periods investigated. In many cases the proportions are actually lower than 1 %. One exception is children from Iran, among whom 2.5 % belonged to the top in 2008–10.

So, what characterises households in which children have a high probability of being at the top of the income distribution? In order to investigate this we specified and estimated logistic regression models in which a position among the 10 % of persons with the highest income in Sweden is the dependent variable. We estimated separate models for children of native born parents and second generation and for each of the periods 1983–85 and 2008–10. Explanatory variables measure location (larger Stockholm, larger Göteborg, larger Malmö, forest counties and other counties), parental education (seven levels), parent’s age (four categories), number of children in the family, age of the child, parents’ country of birth as well as parents’ years since immigration (five or six categories depending on the year). From the estimates we predicted probabilities for native children and for each of the 17 categories of immigrant children and reported them in Table 3. That the immigrant families show great disparity when it comes to levels of education, region in Sweden and number of children is apparent when the descriptions, displayed in the appendix, are studied.

**Table 3:** Predicted probability from logistic regression of belonging in the top 10 % for a child with given characteristics but having parents with different levels of education, country of birth and years since immigration in 1985–87 and 2008–10 (per cent)

	1983–85				2008–10			
Parental country of birth	Both parents have at least 3 years’ post-secondary education		Both parents have at least elementary education but not higher		Both parents have at least 3 years’ post-secondary education		Both parents have at least elementary education but not higher	
SW	37 %		4.4 %		42 %		4.1 %	
	Years since immigration							
	>10	0–5	>10	0–5	>10	0–5	>10	0–5
NO	36 %	21 %	4.5 %	2.2 %	33 %	29 %	2.4 %	1.9 %
GE	28 %	16 %	3.2 %	1.5 %	23 %	20 %	1.4 %	1.2 %
DE	45 %	28 %	3.3 %	1.6 %	42 %	37 %	3.3 %	2.7 %
FI	23 %	13 %	2.5 %	1.2 %	29 %	24 %	1.9 %	1.5 %
GR	16 %	8 %	1.6 %	0.8 %	10 %	8 %	0.5 %	0.4 %
PO	26 %	15 %	2.9 %	1.4 %	16 %	14 %	0.9 %	0.8 %
CH	4 %	2 %	0.3 %	0.2 %	7 %	6 %	0.4 %	0.3 %
FY	19 %	10 %	1.9 %	0.9 %	12 %	10 %	0.6 %	0.5 %
TU	15 %	8 %	1.5 %	0.7 %	13 %	10 %	0.7 %	0.6 %
BO	n.a.	n.a.	n.a.	n.a.	7 %	5 %	0.3 %	0.3 %
LE	4 %	2 %	0.4 %	0.2 %	11 %	9 %	0.6 %	0.5 %
IN	4 %	2 %	0.3 %	0.2 %	9 %	8 %	0.5 %	0.4 %
SY	n.a.	n.a.	n.a.	n.a.	6 %	5 %	0.3 %	0.2 %
IQ	n.a.	n.a.	n.a.	n.a.	5 %	4 %	0.3 %	0.2 %
VI	20 %	11 %	2.1 %	1.0 %	11 %	9 %	0.6 %	0.5 %
EE	8 %	4 %	0.7 %	0.4 %	4 %	4 %	0.2 %	0.2 %
SO	n.a.	n.a.	n.a.	n.a.	2 %	2 %	0.1 %	0.1 %

Source: Logistic regressions estimated by the authors. The predictions are based on a child aged 7–10 years living in a household in Stockholm, consisting of two parents aged 40–49 and one sibling. *n.a.* not available. Abbreviations: *SW* Sweden, *NO* Norway, *GE* Germany, *DE* Denmark, *FI* Finland, *GR* Greece, *PO* Poland, *CH* Chile, *FY* Former Yugoslavia, *TU* Turkey, *BO* Bosnia, *LE* Lebanon, *IN* India, *SY* Syria, *IQ* Iraq, *VI* Vietnam, *EE* Ethiopia and Eritrea, *SO* Somalia

Not surprisingly, Table 3 shows that, it is the level of parental education that is strongly related to the probability of being at the top of the income distribution. We also learn that for a given parental level of education, the probability is typically lower in cases where the parents are foreign born and particularly low if the parents have only recently immigrated. There are also visible differences in probabilities between immigrant children whose parents were born in different countries. Take the example from the period 2008–10 of both parents having at least 3 years of post-secondary education. In cases where the parents are native born the predicted probability of the child belonging to the top 10 % of the population is 42 %. However, if the parents were born in Somalia and have lived in Sweden for at least 10 years, the corresponding probability is no higher than 2 %; if the parents were born in Bosnia, the predicted probability is not more than 7 %; and if the parents were born in Turkey then the probability is no higher than 13 %.^20^

## Rates of Poverty

**Fig. 3:**
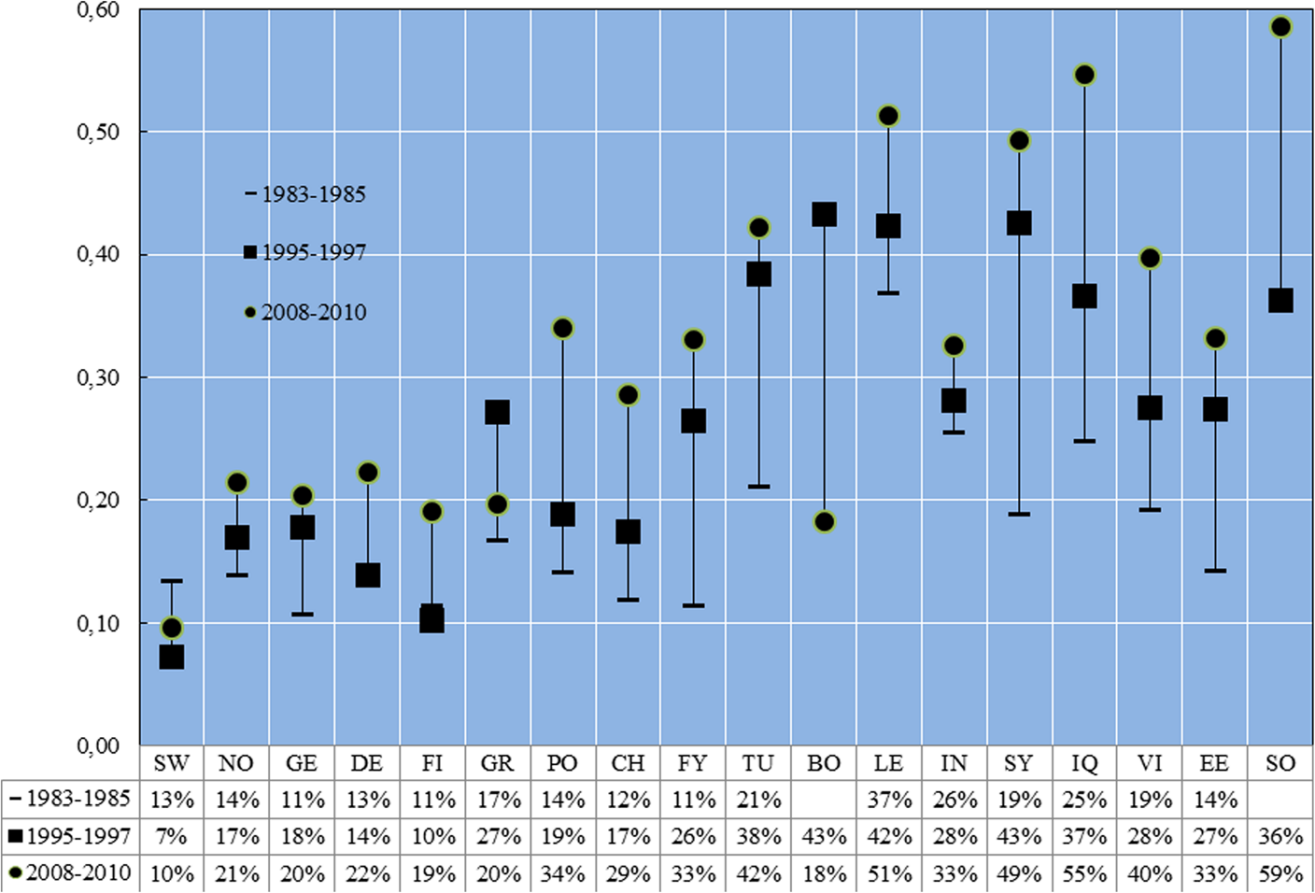
Relative poverty rates for children from 17 different origins and native children 1983–85, 1995–97 and 2008–10 (percent). Source: Authors’ estimates based on data presented in the text. Countries are ranked after the Human development Index. Abbreviations: *SW* Sweden, *NO* Norway, *DE* Germany, *DK* Denmark, *FI* Finland, *GR* Greece, *PL* Poland, *CL* Chile, *FY* Former Yugoslavia, *TR* Turkey, *BA* Bosnia and Herzegovina, *LB* Lebanon, *IR* Iran, *SY* Syria, *IQ* Iraq, *VN* Vietnam, *EE* Ethiopia and Eritrea, *SO* Somalia

Figure 3 shows the relative poverty rates for children belonging to each of the 17 different countries of origin and natives for the three periods. The figure starts with rates of poverty for native children and thereafter orders the countries according to HDI. There are no confidence intervals as the proportions are based on computations for all children living in Sweden, and these do not involve estimates from a sample. Following what is now a common practice when reporting on poverty in member states of the European Union, we define a child as poor in cases where the household equivalent income in which he or she lives is lower than 60 % of the median equivalent household income as observed in the same period in the country. Such a definition means that the real value of the poverty line moves in tandem with median income in society.^21^ In our case the real value of the poverty line increased from 1983–85 to 1995–97, and even more rapidly from 1995–97 to 2008–10.^22^

Starting with children of native born parents we see that the poverty rate stood at 13 % in 1983–85, went down to 7 % in 1995–97 and increased up to 10 % in 2008–10. We have thus found that although we assess a household’s poverty status against a poverty line that increases in real value, poverty among native children was somewhat lower in 2008–10 than in 1983–85. However, the development is the opposite among *all* categories of immigrant children we can follow over both sub-periods: Child poverty rates were higher in 2008–10 than in 1983–85. In some cases, for children whose parents were born in countries with high human development, it means an increase in poverty rates from levels similar to those among majority children in 1983–85 to rates higher than those among majority children. For other categories of immigrant children it means a development from poverty rates being higher than among the majority population to rates being much higher than for the majority population.

Looking in more detail at immigrant children with a background in Bosnia, Somalia and Turkey we see different developments. Poverty rates for children with parents from Bosnia more than halved to come down to 18 % in 2008–10. In contrast the poverty rate among children having parents born in Somalia rose to 59 % while the poverty rate among children having parents born in Turkey went up slightly to 42 %. Children whose parents were from Somalia are not the only one to have very high poverty rates in 2008–10. A majority of children whose parents were born in Iraq or Lebanon were counted as poor during this period, and the same was the case for at least two of every five children whose parents were born in Syria and Vietnam.

What characterises children living in poverty? In order to investigate this we specified and estimated logistic regression models with the status of being poor as the dependent variable. Separate models for 1983–85 and 2008–10 were estimated for immigrant and children with native born parents. Explanatory variables measure location (larger Stockholm, larger Göteborg, larger Malmö, forest counties and other counties), parental education (seven levels), parents’ age (four categories), number of children in the family, age of the child, parents’ country of birth as well as parents’ years since immigration (four categories for 1983–85 and five categories for 2008–10 categories). From the estimates we predicted probabilities for native children and for each of the 17 categories of immigrant children and reported them in Table 4.

**Table 4:** Predicted probability from logistic regression of being poor (60 % of median poverty line) for a child with given characteristics but having parents with different levels of education, country of birth and years since immigration in 1985–87 and 2008–10 (per cent)

	1983–85				2008–10			
	Both parents have at least 3 years’ post-secondary education		Both parents have at least elementary education but not higher		Both parents have at least 3 years’ post-secondary education		Both parents have at least elementary education but not higher	
SW	2 %		12 %		2 %		22 %	
	Years since immigration							
	>10	0–5	>10	0–5	>10	0–5	>10	0–5
NO	7 %	10 %	14 %	20 %	7 %	22 %	25 %	56 %
GE	7 %	10 %	14 %	20 %	7 %	22 %	25 %	56 %
DE	6 %	9 %	13 %	19 %	9 %	26 %	29 %	61 %
FI	5 %	7 %	10 %	15 %	7 %	23 %	26 %	57 %
GR	6 %	9 %	13 %	19 %	8 %	24 %	27 %	58 %
PO	6 %	9 %	13 %	19 %	10 %	30 %	34 %	66 %
CH	7 %	11 %	3 %	5 %	12 %	33 %	37 %	68 %
FY	5 %	7 %	10 %	15 %	8 %	25 %	29 %	60 %
TU	5 %	7 %	10 %	15 %	15 %	39 %	43 %	74 %
BO	n.a.	n.a.	n.a.	n.a.	5 %	17 %	20 %	48 %
LE	22 %	30 %	11 %	16 %	17 %	44 %	48 %	77 %
IN	10 %	14 %	20 %	28 %	16 %	41 %	46 %	76 %
SY	4 %	6 %	9 %	14 %	19 %	47 %	52 %	80 %
IQ	8 %	15 %	16 %	23 %	19 %	47 %	52 %	80 %
VI	3 %	4 %	6 %	9 %	12 %	34 %	38 %	69 %
EE	4 %	6 %	9 %	13 %	10 %	29 %	33 %	64 %
SO	n.a.	n.a.	n.a.	n.a.	14 %	38 %	42 %	73 %

Source: Logistic regressions estimated by the authors. The predictions are based on a child aged 7–10 years living in a household in Stockholm, consisting of two parents aged 40–49 and one sibling. *n.a.* not available. Abbreviations: *SW* Sweden, *NO* Norway, *GE* Germany, *DE* Denmark, *FI* Finland, *GR* Greece, *PO* Poland, *CH* Chile, *FY* Former Yugoslavia, *TU* Turkey, *BO* Bosnia, *LE* Lebanon, *IN* India, *SY* Syria, *IQ* Iraq, *VI* Vietnam, *EE* Ethiopia and Eritrea, *SO* Somalia

The predictions reported in Table 4 show that the probability of being poor for a child was as low as 2 % in 1983–85 as well as in 2008–10 if both parents were native born and had at least 3 years of post-secondary education. The predicted probability was considerably higher for parents who had a short education, particularly in 2008–10 when the probability reached 22 %.^23^ How long the parents have lived in Sweden also makes a difference to the predicted probabilities of being poor. The predicted probability of being poor was notably high in 2008–10 if the parents had only primary school education and were newly arrived. In such cases the predicted poverty probability typically reached above 50 % and in cases of parents born in Syria or Iraq much higher than 50 %. It is worth noting that children having parents born in Somalia did not stand out once we controlled for parental characteristics, as these children had predicted poverty rates that were comparable to those of several other background countries.^24^

**Fig. 4:**
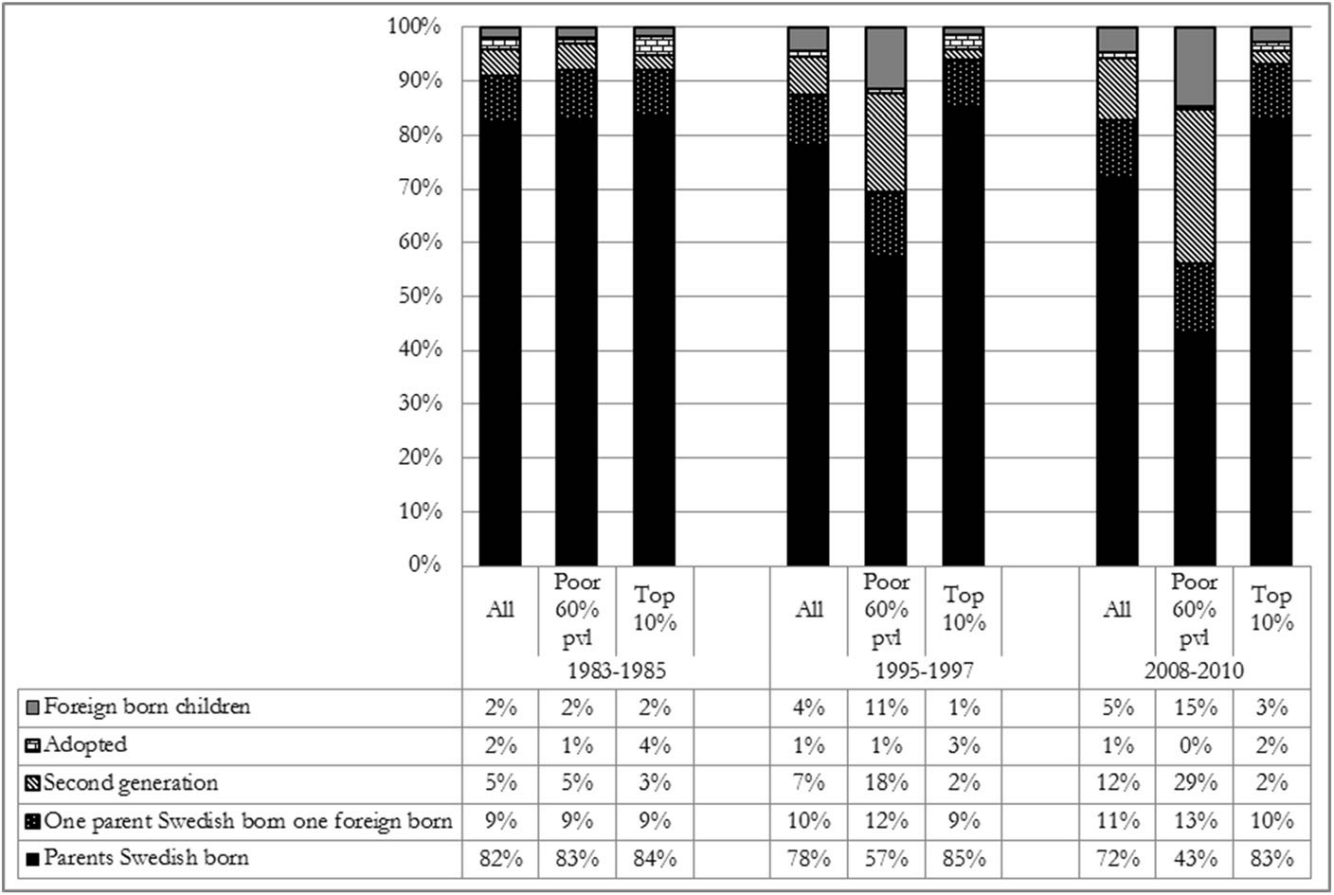
Composition of children by migration status in the total population, at the top and among the poor 1983–85, 1995–97 and 2008–10

Before summing up this study in the next section it might be useful to show the composition of children at the bottom and top of the income distribution in Sweden by country of background during the three periods under study in Fig. 4. In the figure we include not only the second generation immigrant children we have studied above, but also children born abroad, children born in Sweden with one foreign-born parent and one native-born parent, and adopted children. We see that in 1983–85 immigrant children were represented at the top and at the bottom similarly to their presence among the total population of children in Sweden. Thereafter their representation among the poor increased, and in 2008–10 immigrant children were in majority of all poor children. In contrast, immigrant children at the top of the income distribution in 2008–10 were in the minority, similarly to how they were represented in 1983–85.

## Conclusions

In this article we have studied how immigrant children in Sweden are faring by analysing income-based child indicators. We have focused on 17 categories of children themselves born in Sweden to parents who were born in various countries. By studying the three periods 1983–85, 1995–97 and 2008–10, we could capture the large changes that took place in many dimensions over this generation-long period. For example, Sweden changed from a country with low unemployment to one that in this respect became more similar to other European countries. Over the period income grew more slowly in the lower segment of the income distribution than in the higher segments and the composition of the immigrant children population changed drastically. The previously large population of children with a background in Finland diminished rapidly in number while instead the number of immigrant children with a background in distant countries with low or relatively low human development increased. At the end of the period, children with a background in Iraq were the single largest category of immigrant children.

We found that in 1983–85 there were very small gaps in mean income between majority children and children with parents born in high income countries, while we observed more of an income disadvantage for children with parents born in a country with low or middle human development. However, the relative position of immigrant children of *all* backgrounds weakened from 1983–85 to 1995–97 for second generation immigrant children as well as for children with one foreign born and one native born parent. This occurred as the labour market situation of immigrant parents deteriorated more than among majority parents. During the period 1995–97 to 2008–10 changes of gaps in average incomes between native children and various categories of immigrant children shows a more complex picture. Most gaps towards natives for children with two foreign born parents widened, but not as rapid as during the first period. In addition among children with one foreign born parent and one native born parent there were not more examples of increasing gaps as of the opposite.

This study has also reported a large heterogeneity across 17 different backgrounds of immigrant children in income-based indicators. To a large extent it is a variation along the dimension the degree of human development in the country where the parents were born. We have reported that children born in Sweden by parents from Denmark, Germany or Norway, all countries with a high degree of human development, were as likely as majority children to be observed at the top of the income distribution, which is in contrast to children of parents from countries with middle or low human development.

We have also reported that poverty rates of immigrant children in 2008–10, were much higher among for children with parents born in countries with middle or low human development. At an extreme a majority of children whose parents were born in Iraq, Lebanon and Somalia were counted as poor, and the same was the case for two out of five children with parents born in Turkey, Syria or Vietnam. However, we also showed that poverty rates changed differently between 1995–97 and 2008–10 for different categories of children having parents born in countries with middle and low human development: Poverty rates among Bosnia children more than halved as increasingly many parents found a job, poverty rates among Turkish children were roughly constant and poverty rates among Somali children increased rapidly.

Over the period here studied has child poverty in Sweden become a problem very much linked to immigrant children. Our results show that a majority of children living in Sweden that are classified as poor in 2008–10 were immigrant children of various categories (foreign born, second generation immigrants, or of mixed background).

The high poverty rates among categories of immigrant children should primarily be seen in the light of the difficulties of their parents finding employment, which in turn is linked to being newly arrived and having a short education. The results from the regression models indicate that, to some degree, cross-country-of-origin differences in income-based child indicators can be attributed to the reasons and qualifications the parents had when entering Sweden and the number of years that have elapsed since their immigration. Finally, one should also understand that social and economic policy in general has a role for how gaps in child poverty rates between natives and immigrant children develop. For example child allowances, unemployment benefits and some other benefits are on average more important component of the family budget for immigrant families with children than for the naive counterparts. The value of such benefits did not change as rapidly as earnings from the end of the 90s to 2008. From this follows that gaps in poverty rates between immigrant children and native children would in 2008–10 have been smaller if for example child allowances had since the beginning of the beginning of the new Millennium been indexed to wages.

## Appendix

(Table 5).

**Table 5:** Average equivalent disposable income among native children and children with mixed background from 17 different origins 1983–85, 1995–97 and 2008-20

Parental country of birth	Average income 1983–85 SEK	Average income 1995–97 SEK	Average income 2008–10 SEK	Average income 1983–85 as fraction of native income	Average income 1995–97 as fraction of native income	Average income 2008–10 as fraction of native income	Growth rate 1995–97 compared to 1983–83 %	Growth rate 2008–10 compared to 1995–97 %
Sweden	113589	148362	235733	100 %	100 %	100 %	31 %	59 %
Norway	111437	143488	227659	98 %	97 %	97 %	29 %	59 %
Germany	120149	145511	236217	106 %	98 %	100 %	21 %	62 %
Denmark	111590	142106	230099	98 %	96 %	98 %	27 %	62 %
Finland	110720	142848	221763	97 %	96 %	94 %	29 %	55 %
Greece	100794	125666	209543	89 %	85 %	89 %	25 %	67 %
Poland	114621	141155	208558	101 %	95 %	88 %	23 %	48 %
Chile	102617	122843	182746	90 %	83 %	78 %	20 %	49 %
Former Yugoslavia	104631	129708	193233	92 %	87 %	82 %	24 %	49 %
Turkey	103054	113276	165863	91 %	76 %	70 %	10 %	46 %
Bosnia		110262	182364		74 %	77 %		65 %
Lebanon	94416	108901	167503	83 %	73 %	71 %	15 %	54 %
Iran	102604	108134	203266	90 %	73 %	86 %	5 %	88 %
Syria	106327	93976	173332	94 %	63 %	74 %	−12 %	84 %
Iraq	113565	99865	156244	100 %	67 %	66 %	−12 %	56 %
Vietnam	102950	108685	186923	91 %	73 %	79 %	6 %	72 %
Ethiopia, Eritrea	112955	106784	193785	99 %	72 %	82 %	−5 %	81 %
Somalia		99537	145607		67 %	62 %		46 %

## Footnotes

1 Examples of such country overviews are Katz and Redmond (2010) for Australia, Wanner and Fibbi (2010) for Switzerland, Clauss and Nauck (2010) for Germany, De Valk (2010) for the Netherlands, Mencarini (2010) for Italy and Crawley (2010) for the United Kingdom.

2 As income for a particular household often changes a fair amount from 1 year to another we use 3-year periods in our analyses of income based child indicators.

3 Our calculations are based on the microdata presented in Section 4.

4 Other categories of immigrant children are (according to our data) 84 000 foreign born and 184 000 born in Sweden having one foreign and one native-born parent. In addition there are 15 000 adopted children of whom most are foreign born.

5 For a survey on the labour market situation of adult immigrants see Bengtsson et al. (2005). Obucina (2013) and Kesler (2014) investigated aspects of adult immigrant poverty in Sweden.

6 See the Ministry of Health and Social Affairs (2002) and references therein for development during the 1990s.

7 See, for example, Edin and Åslund (2001), Gustafsson et al. (2004) and Eriksson (2011).

8 Factor income is defined as income from employment and capital but not including transfer payments and income taxes.

9 A reason that still applies is the increased importance of capital incomes, which are concentrated at the top of the income distribution. See also Björklund and Jäntti (2013) and Fritzell et al. (2014).

10 We have chosen 17 background countries where at least 5 000 children were found in one of the periods of observation. Should a child have parents born in different foreign countries, for the purpose of this study we assign it the father’s country of birth. A person born in present-day Bosnia and who entered Sweden when Yugoslavia existed is classified as born in (former) Yugoslavia. Pay attention to that in our classification does the citizenship of a person not play a role.

11 On the labour market situation of adult migrants from Somalia and Turkey during the new millennium see Bevelander and Dahlstedt (2012). See Ekberg and Ohlsson (2000) for the remarkable rapid labour market progress among some immigrants from Bosnia.

12 For example in the Swedish setting there are attempts to collect information on various aspects of a child directly from the child, see Jonsson and Österberg (2010).

13 For details of the equivalence scale see the documentation for the LISA database (Statistics Sweden 2011).

14 Only children who could be observed during all 3 years of observation are included; that is, children that are born or become adults, die or emigrate during the 3-year period of observation are not included.

15 We deleted children from the data with parents born in Denmark living in the county of Skåne with a zero income. It is most probable that those parents were commuting to a job in Denmark and that their earnings were not included in the Swedish tax register.

16 See http://hdr.undp.org/en/data.

17 But it is worth nothing that the average number of years in the country is comparable for parents to Somalian and Bosnian children (12.5 years on average in 2010 for the first group and 14.5 years for the second).

18 It is beyond the scoop of this paper to analyze reasons for the differences in child income between second generation children and children in mixed families.

19 Most probably the decreased representation of children with Polish background at the top of the distribution can be attributed to changed composition of Polish immigrants to Sweden. Many Polish immigrants who arrived to Sweden from the end of the 40s and decades thereafter were refugees or came for family reunion, many having a longer education. When Poland became a member of EU in 2004 a flow of work migrants with a more mixed education background arrived in Sweden.

20 When we relate the coefficients for the country of background dummies to the index of human development we find a positive relation for both years. R^2^_adj_ = 0.382 in 1995–97 and R^2^_adj_ = 0.798 in 2008–10.

21 The 60 % poverty line for the three periods of income in 2011 prices was (for one person) 117 312 in 1983–85, 143 408 in 1995–97 and 209 255 in 2008–10. In 2009 did 1 SEK equal 0.094 €.

22 If we had chosen to use a poverty line that represents the same purchasing power (using the consumer price index) for all years as the one we applied for 1983–85 it would have led to lower poverty rates for all categories studied here for the period 2008–10. For example, in 2008–10 as few as 1 % of majority children would have been counted as poor, and the maximum in the same period would have been 11 % (for children whose parents were born in Syria).

23 It should be understood that the number of majority parents having a short education was considerably smaller in 2008–10 than in 1983–85.

24 When we relate the coefficients for the country of background dummies to the index of human development we find no indication of a relationship in 1995–97 (R^2^_adj_ = 0.008) but a negative one in 2008–10 (R^2^_adj_ = 0.215).
